# Is Digital Treatment the Holy Grail? Literature Review on Computerized and Blended Treatment for Depressive Disorders in Youth

**DOI:** 10.3390/ijerph17010153

**Published:** 2019-12-24

**Authors:** Sanne P. A. Rasing, Yvonne A. J. Stikkelbroek, Denise H. M. Bodden

**Affiliations:** 1 Child and Adolescent Studies, Utrecht University, P.O. Box 80140, 3508 TC Utrecht, The Netherlands; y.stikkelbroek@uu.nl (Y.A.J.S.); d.bodden@uu.nl (D.H.M.B.); 2 Child and Adolescent Psychiatry, GGZ Oost Brabant, P.O. Box 3, 5427 ZG Boekel, The Netherlands; 3 Developmental Psychopathology, Radboud University, P.O. Box 9104, 6500 HE Nijmegen, The Netherlands

**Keywords:** depression, adolescents, youth, computerized, blended, treatment, review

## Abstract

Computerized and blended treatments seem to be an attractive treatment for adolescents as an alternative to face-to-face treatment, but mental health professionals seem hesitant to use these treatment modalities. This review provides an overview of factors contributing to and withholding from using computerized or blended treatment in routine care. Three databases were searched with terms related to (1) adolescents, (2) depression, (3) computerized or blended, and (4) treatment. Of the 33 articles identified, 10 focused on unguided computerized treatments, six on guided, two on blended, two compared unguided, blended- and face-to-face treatment to no treatment, and eight studies on games. Further, two articles that were focused on an online monitoring tool and three on intervention characteristics or preferred modes of help-seeking. Evidence for effectiveness, adherence, drop-out, and forming therapeutic relations were suspected to be barriers, but are no reason to reject computerized or blended treatment. Improvement in mental health literacy and the possibility to tailor the intervention are facilitators. However, adolescents’ intention to seek help, acceptability of computerized treatment, symptom severity, time spent by therapist, and other facilities are identified as barriers and they need to be taken into account when using computerized or blended interventions. Nevertheless, computerized and blended are promising treatments for depressed youth.

## 1. Introduction

Depressive disorders are among the most prevalent mental health disorders in youth and they are a major contributor to disability [1,2]. Before the age of 19, more than 15% of youth has suffered from at least one depressive episode in their life [3]. Suffering from depression at a young age increases the risk of social problems, poor academic and professional performance, school drop-out, and health problems, such as substance abuse, comorbid disorders, and suicide [4,5,6]. Besides, a high risk of recurrence and chronicity characterize depressive disorders [7,8]. Given these consequences, treating the disorder effectively at an early age is crucial.

Cognitive Behavioural Therapy (CBT) and Interpersonal Psychotherapy are the first choices of treatment from the current available forms of psychotherapy for depressed youth [9,10,11]. Nevertheless, the average effect size of CBT as compared to a variety of control conditions (i.e., active treatments targeting non-depressive symptoms, active treatment for depression, medication placebo) is small to moderate (ES = 0.34–0.80) [12,13], and only 50% of the treated youth show symptom improvement after psychotherapy [14]. This shows that face-to-face treatment is effective in treating depression, but certainly not for all depressed adolescents. Above all, the number of youth that drop out of treatment is high; approximately 50% (with a range from 17 to 72%) of all adolescent outpatients drop out of treatment according to a meta-analysis [15]. Important barriers for youth seeking help or treatment that are often mentioned are lack of accessibility, for example lack of time, high costs, or no transport [16]. This strongly suggests that more attractive and more suitable treatments are needed, especially for adolescents who do not prefer to visit a mental health professional each week. In doing so, individual outcomes could be improved. 

Computerized treatments seem to be an obvious solution to several problems, because of their availability, anonymity, and accessibility [17,18]. Treatment through computer-based programs (for example CD-Roms) and treatment through internet-based programs using online applications (for example email or web-based programs), that are often the most recently developed programs define computerized treatment programs. In this review article, computer-based and internet-based programs will both be conceptualized as computerized programs. Computerized treatments can be unguided, meaning that patients work through the program themselves, or guided, meaning that there is support of a therapist. Characteristic of these guided programs is that communication between patient and support is not synchronized, which means that patients and therapists communicate not at the same time [19]. Treatment programs can also be presented as a game, where the content of the treatment is presented in a gamified manner [20]. Earlier research has demonstrated that computerized treatment is moderately effective in treating depressive disorders in youth when compared to no treatment and waiting list controls [18]. A meta-analysis of treatment effects in depressed adults showed equivalent effects of guided internet-based treatment when compared to face-to-face treatment [21]. However, another meta-analysis has demonstrated that computerized CBT for adults in clinical practice, i.e., outside controlled research settings, has smaller effects and a higher attrition rate when compared to computerized CBT in adults who are recruited for participation in research [22]. A recent development is combining computerized treatment with face-to-face treatment into the so-called blended treatment in order to overcome the downsides of computerized treatment [23]. In blended treatment, patients work independently while using computerized content and receiving guidance by their therapist by means of personal face-to-face contact [23,24]. Blended treatment specifically aims to preserve the therapeutic relation [25]. In such a way, blended treatment contains the benefits of both face-to-face treatment as well as computerized treatment. 

Yet, professionals in youth clinical practice still hold a skeptical attitude towards computerized and blended treatments [26,27]. They doubt the effect of these treatment modalities [28] and they assume that there is a high risk of adverse events [19], such as an increase in symptom severity and suicidal ideation, as a consequence of fewer or face-to-face treatment sessions. As a result, professionals are hesitant to use these treatment modalities [29]. The frequently mentioned arguments against solely using computerized or blended treatments include difficulties in monitoring the increase of symptom severity and the risk for suicide [19], no match with the needs of the adolescent, low treatment adherence, and high drop-out. As a consequence, therapists often refrain from using computerized or blended treatments and continue to use face-to-face treatments [28]. However, the evidence for not using these treatments is unclear, since the potentially facilitating factors (i.e., availability, anonymity, accessibility) and barriers (i.e., effect, drop-out) to use computerized or blended treatments in treating depressed youth are not systematically described. 

The aim of this review is to provide an overview of the knowledge on factors that could contribute to, or withhold from, using computerized or blended treatment in routine care for adolescents with depression. We will review whether intervention effects, mental health literacy, adherence and drop-out, intention to seek help, tailoring the intervention, symptom severity, risk monitoring, acceptability, treatment engagement, time spent by therapist, therapeutic relation, and other facilities for computerized or blended interventions are facilitators or barriers in the use of treatment of depressed adolescents. Additionally, suggestions for future research are given.

## 2. Method

### 2.1. Search Strategy

Databases Medline, PsycInfo, and Embase were systematically searched in January 2019. The search terms were (1) adolescent OR adolescence OR young adult OR teen, AND (2) depression OR depressive OR depressed OR MDD OR mood disorder OR dysthymic OR dysthymia, AND (3) blended OR supported OR guided OR assisted OR computerized OR computerised OR computer-based OR internet-based, AND (4) treatment OR therapy OR intervention OR program. 

We identified 1686 studies while using these terms. Besides these, 23 studies were identified through other sources, for example through hand searching reference lists of relevant studies and reviews. In total, we identified 1709 studies. After removing 927 duplicates, 782 studies remained. The first author screened the first, titles, and abstracts to determine whether studies were relevant for the review. The inclusion criteria for the current review were that (1) the presented interventions were computerized or blended, (2) interventions were used as treatment (not as prevention), (3) were directed at depressive symptoms or disorders, (4) were aimed at youth (i.e., aged 12–23 years), (5) were published in peer-reviewed journals in the English or Dutch language, and (6) contained original data. This resulted in excluding 724 studies, because the studies were not about computerized or blended programs (*k* = 187), did not involve treatment (but were, for example, on online diagnostic tools or online learning environments) (*k* = 167), were not directed at depressive symptoms or disorders (*k* = 334), were not aimed at adolescents or youth (*k* = 18), were not written in the English or Dutch language (*k* = 4), or did not contain original data (but were, for example, reviews or meta-analyses) (*k* = 14); as a result, 58 studies remained.

**Figure 1:**
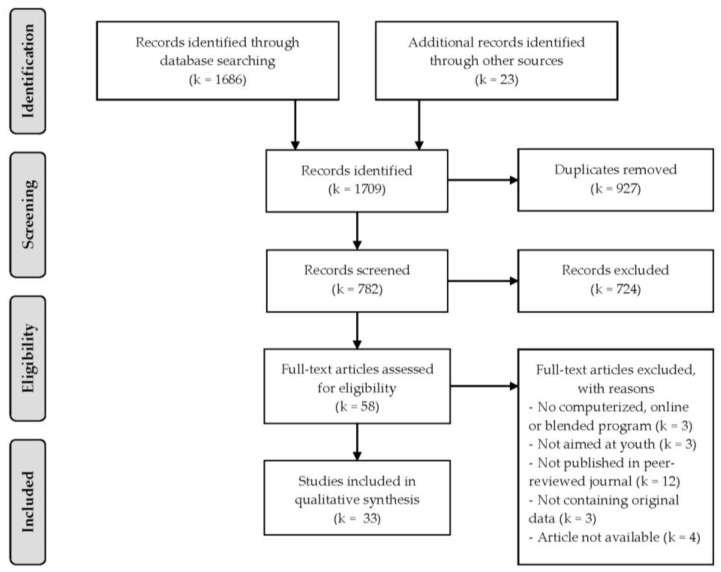
Flow chart of study selection.

Next, titles, abstracts, and method sections were systematically reviewed and considered for inclusion. Of the 58 remaining studies, 25 studies were excluded because they were not about computerized or blended interventions (*k* = 3), not aimed at youth (*k* = 3), were not published in a peer-reviewed journal (but were, for example, conference abstracts) (*k* = 12), did not contain original data (but were, for example, published study designs) (*k* = 3), or were not available (*k* = 4). Figure 1 presents an overview of the final selection of 33 studies. These studies were used in a qualitative analysis on barriers and facilitating factors (including effectiveness) that play a role in using these treatment modalities in the treatment of depressed youth. 

### 2.2. Data Extraction

The following data could be extracted from the included studies: intervention effects, mental health literacy, adherence and drop-out, intention to seek help, tailoring the intervention, symptom severity, risk monitoring, acceptability, treatment engagement, time spent by therapist, therapeutic relation, and facilities for computerized or blended interventions. For these factors, we will describe whether studies show whether these factors are actual barriers or facilitators in the use of treatment of depressed adolescents.

## 3. Results

### 3.1. Description of Studies

Table 1 presents details of the included studies (*k* = 33). Sixteen studies were aimed at computerized treatments, of which nine were unguided computerized treatments (four computer-based and five internet-based) [30,31,32,33,34,35,36,37,38] and seven studies were guided computerized treatments (one computer-based and six internet-bases) [39,40,41,42,43,44,45], two studies at blended treatment [46,47], two studies compared unguided computerized, blended- and face-to-face treatment to no treatment [48,49], and eight studies were aimed at self-help internet-based games [50,51,52,53,54,55,56,57]. Furthermore, two studies that were focused on the use of an online monitoring tool aimed at registering and monitoring treatment progression [58,59] and three studies were aimed at characteristics of online interventions or studied the preferred modes of help seeking [60,61,62]. 

Fourteen studies were mainly focused on the evaluation of the effects of the intervention. In these studies, the designs varied between randomized controlled trials (*k* = 10) [34,35,36,38,39,44,45,49,52,57], pre-post designs with control group (*k* = 2) [33,42], and pre-post designs without control group (*k* = 2) [30,40]. Sixteen studies were mainly focused on the feasibility, usability, and acceptability of computerized and blended interventions. The study designs were mixed and they contained qualitative studies with focus groups of patients, healthy adolescents or mental health professionals (*k* = 6) [41,53,54,56,58,59], randomized controlled trials (*k* = 3) [43,47,48], pre-post designs without control group (*k* = 5) [31,46,50,51,55], and cross-sectional studies (*k* = 2) [32,37]. Three studies were mainly focused on the preferences of patients regarding treatment modalities [32,60,62].

### 3.2. Intervention Effects

The evidence shows that participants who received unguided computerized CBT (cCBT) less often showed a depressive disorder after receiving the intervention than before [30]. Further, participants showed an increase in perceived control after receiving unguided cCBT [33]. When unguided cCBT was compared to a wait list condition, the findings showed that depressive symptoms decreased significantly more in the unguided CBT condition [34]. The results also showed that the intervention was equally effective for boys and girls [35]. The findings of two studies showed that the decrease of depressive symptoms was larger in the participants in the unguided CBT condition when compared to the participants in the psychoeducational control condition [36,38]. Changes in mental health literacy after unguided computerized treatment were not reported.

After receiving guided cCBT participants showed less depressive symptoms [40]. Depressive symptoms were significantly lower after 10 weeks in the guided cCBT condition when guided cCBT was compared to a waitlist condition [51]. When only including boys, the findings showed no difference in the reduction of depressive symptoms between guided cCBT and psychoeducation [42]. When guided cCBT was compared to treatment as usual, a larger symptom reduction was found in the participants who received guided CBT [43]. Additionally, guided CBT has more benefits for depressed adolescents than the waitlist condition [35,45]. Adolescents showed an increase in literacy and confidence in their knowledge on depression, independent from changes in depressive symptoms after receiving guided cCBT [39].

Blended treatment and treatment as usual showed equal effects in a decrease of depressive symptoms [47]. Moreover, adolescents who received blended CBT for their depressive disorder showed a significant increase in knowledge of CBT concepts after the intervention [47]. Two other studies showed that blended CBT was more effective in treating depressive symptoms, as compared to unguided cCBT alone and face-to-face CBT alone. Nonetheless, participants in all three conditions showed a significant decrease in depressive symptoms when compared to participants without an intervention [48,49]. It needs to be noted that participants in the latter two studies were young adults between 18 and 25, and cannot be seen as a representation of patients in their adolescence.

When the adolescents participated in a self-help CBT-based computer game, their depressive symptoms decreased significantly [55]. However, a decrease of depressive symptoms was not significantly different from adolescents who received treatment as usual [57]. Another study showed that depressive symptoms decrease more after a self-help CBT-based computer game (78%) when compared to a wait list condition (36%) [52]. Two of the online modules of the self-help CBT-based computer game Sparx aimed at improving mental health literacy and the knowledge on depression (e.g., “Learning about depression” in Sparx) were rated as the most useful modules [55].

### 3.3. Adherence and Drop-Out

Adherence is often considered to be lower and drop-out higher in computerized treatments as compared to face-to-face treatments [62], hence they are considered to be barriers. Between 62% and 94% of the participants completed (almost) the entire unguided cCBT [36,38]. Other findings show that between 0% and 21% of participants dropped out of unguided cCBT [34,38,48].

For guided cCBT, Van der Zanden, Kramer, Gerrits, and Cuijpers [45] reported that only 20% of the participants completed the complete program and attended all sessions, which was exceptionally low when compared to the other studies. However, each session lasted 90 min. and more than half of the participants attended four of the six online sessions. Other studies found that between 61 and 70% completed the full program [40,44]. Johnston, Dear, Gandy, Fogliati, Kayrouz, Sheehan, Rapee, and Titov [40] also reported that 17% of the participants dropped out. It was also mentioned that some individuals did not complete the intervention, but the reason for doing so was not because of dissatisfaction with the intervention [43].

Adherence was not reported for blended interventions. The drop-out varied between studies: De Vos, Tromp, Bodden, and Stikkelbroek [46] reported that 13% of the patients dropped out, Kobak, Mundt, and Kennard [47] reported 10%, while Sethi [48] reported no drop-outs.

For games, it was found that between 60% and 81% of the participants completed the entire self-help CBT-based computer game Sparx [55,57]. Another 9 to 26% completed four to six modules of the game. However, when the self-help CBT-based computer game was used for adolescent inpatients, only 10% completed the intervention [50]. Fleming, Dixon, Frampton, and Merry [52] reported that there was no difference in proportion of drop-outs between a self-help CBT-based computer game condition and the waitlist condition. 

### 3.4. Intention to Seek Help

In general, a low intention to seek help characterizes depressed adolescents [52]. Indeed, it was found that depressed adolescents are more inclined to avoid seek help than to get face-to-face or computerized treatment [60]. After following an unguided computerized program for depression, adolescents are more intended to seek additional mental health treatment [33,60]. Higher mental health literacy indirectly predicted the intention to seek computerized or face-to-face treatment. Although computerized programs (i.e., unguided or guided) seem to be more available and accessible due to more privacy for example, adolescents report that they need to be motivated to use computerized programs and need to be aware of the existence of computerized treatments and where to find them [31].

### 3.5. Tailoring the Intervention

The possibility to adapt an intervention to the individual preferences, needs, and goals is called tailoring interventions [63]. Computerized interventions are often considered easier to tailor, and this can be seen as a facilitator of computerized interventions. Adolescents reported that computerized interventions are accessible at any time and place and can, therefore, be easily tailored to individual preferences [31]. Moreover, a study reported that study participants found it appealing that several computerized programs allowed for patients to tailor exercises and create their own treatment goals, for example by creating their own behavioral activation plan [32]. 

### 3.6. Symptom Severity

The severity of depressive symptoms or having a severe depressive disorder has been described as a barrier to use computerized treatment. The perception of mental health professionals is that online interventions, regardless of being unguided, guided, or blended, are not adequate for adolescents with severe depressive symptoms or a major depressive disorder, because the computerized support system is not suitable [41]. Additionally, during computerized treatment, the severity of depressive symptoms can be a barrier for proceeding. An increase in depressive symptoms during the intervention was a reason to change the computerized intervention into a face-to-face treatment, because mental health professionals believed that the online intervention was inappropriate when the depression became too severe [61].

Additionally, it was found that the use of this app decreased when mental health improved in testing the feasibility of an online application aimed at registering and monitoring treatment progression and to access crisis plans. The app became less useful when treatment was finished or almost finished, because the need to access crisis plans and update treatment progression was smaller [58]. 

### 3.7. Risk Monitoring

The concern of mental health professionals is that they are not able to monitor risk of self-harm and suicidal ideation in the computerized treatment of depressive disorders [59]. Mental health professionals reported that it was difficult to decide which notifications were actually alarming in the adolescent’s condition and needed interference when guided computerized interventions were used [41]. 

Another concern is that treatment activity of the patient is often not synchronous with the activity of the therapist. Consequently, the therapist is not always able to monitor progress and risk. Clinicians participating in a qualitative study suggested that other people in mental service could check for alerting messages [59].

### 3.8. Acceptability

Participants that used unguided cCBT rated the program as useful and helpful [32]. Most of the participants would recommend it to others [33,36]. One study asked participants to rate the actual website. The participants were positive and they reported that it was easy to use and relatable [33]. Unguided cCBT allows for privacy, which participants rated as very important [31]. The strongest predictor for use and non-use appeared to be gender, as females are more likely to use unguided cCBT [37]. Guided cCBT was evaluated by participants as useful and ‘worth their time’ [40]. Most of the participants were satisfied [40,43] and they rated the treatment as acceptable and would recommend it to friends [40].

Blended CBT was rated as flexible, helpful, and easy to use at a location the adolescent choose, which is important, because adolescents prefer not to visit mental health services for face-to-face treatment [46,47]. However, more than half of the participants would not recommend it to others. Improvements in depressive symptoms were attributed to the face-to-face sessions and not to the computerized program [46]. Further, adolescents reported that the therapists’ messages were useful and motivating [46].

Participants reported that the computer game Sparx was acceptable, useful, and feasible as intervention [50,53,55]. They also reported that the program was appealing for young people [55,57], although the generic version of Sparx did not specifically address topics for sexual minority youth (i.e., youth attracted to the same sex, both sexes, or not sure of their sexual attraction) [54]. The majority of adolescents would recommend Sparx to others [55,57]. Privacy can be easily guaranteed [55] and, for members from a small community, it seems to be a promising way to seek help [51,56]. Nonetheless, internet safety needs to be considered by the users [56].

Adolescents were positive about an online application aimed at registering and monitoring to enhance the effects of cCBT, because it was easier to contact mental health professionals [59]. Patients and mental health professionals were both positive about the possibilities of an online monitoring tool for treatment progression and access crisis plans to fit well with the treatment [58].

Besides the acceptability of specific interventions, registered in earlier mentioned studies, two studies explicitly focused on modalities of treatment delivery and preferences of adolescents and young adults. The first study revealed that only a minority (16%) of the Australian adolescents preferred computerized treatment. Two-thirds of the adolescents preferred face-to-face treatment; however, the highest percentage of adolescents had no intention to seek help at all [60]. Another study revealed that the majority of patients of 18 years and older preferred face-to-face treatment, and there was also preference of a combination of face-to-face and computerized treatment (blended) over complete computerized treatment [62]. It needs to be noted that participants in this latter study are aged 18 and older, and cannot be seen as a representation of patients in their adolescence. 

### 3.9. Treatment Engagement 

The low treatment engagement of youth in computerized programs is often mentioned as a barrier. Patients and mental health professionals reported that an online application (a so-called electronic personal health record) aimed at registering and monitoring treatment progression was stimulating adolescents’ engagement in a hospital-based outpatient treatment [58]. In addition, an online behavioral activation tool was assessed by youth to have a motivational effect on treatment engagement [32]. No studies reported results on treatment engagement of adolescents or therapists for computerized treatment (unguided or guided), blended treatment, or self-help game.

### 3.10. Time Spent by Therapist

The time investment of therapists is seen as a facilitator in using computerized treatment, because it is often estimated that the time spent by therapists for guided computerized treatment or blended treatment is less than for face-to-face treatment [64,65]. One of the studies included in this review registered the time that mental health professionals spent on each patient who received guided cCBT. On average, they spent more than 45 min. per week per patient. Besides the chat sessions, approximately 30 messages were sent to each patient [44]. Mental health professionals reported that an online monitoring tool (to monitor mood, risk, and treatment adherence of adolescents completing a cCBT program) was not fully integrated in the treatment and they perceived the monitoring tool as an additional task. The monitoring took more time than anticipated and not all professionals used it [59]. 

### 3.11. Therapeutic Relation

Mental health professionals often express their concerns regarding the quality of the therapeutic relation when solely using a computerized treatment, hence making it a barrier to use computerized treatment programs. They were concerned whether using a computerized program could establish the same relationship as having face-to-face sessions [59]. However, mental health professionals who already used a computerized program, more specifically guided cCBT, reported that it did not negatively impact their therapeutic relationship with the depressed adolescent [43,44,59]. Guided computerized treatment could even support the therapeutic interaction, because the adolescents learn how to express themselves in the computerized program [47,61]. An online application that was aimed at registering and monitoring treatment progression and accessing crisis plans was reported to facilitate communication. This resulted in a positive relation between therapist and patient, because the communication during the face-to-face sessions was focused on treatment techniques and not on monitoring [58].

### 3.12. Facilities for Computerized Interventions 

Another important barrier for using guided or blended interventions is a lack of training in the computerized part of the treatment protocol. Mental health professionals did not receive any training or the training did not met their needs [61]. Further, it was mentioned that the internet connection was to slow for the use of internet interventions, especially in clinical settings [61].

## 4. Discussion

The aim of this review was to provide an overview of the knowledge of factors that could contribute to, or withhold from, using computerized or blended treatment in routine care for adolescents with depression. We expected that acceptability, low intention to seek help, the possibility to tailor the intervention, and time investment of therapist are considered as facilitators for the use of computerized interventions. On the other hand, we expected that limited evidence for effectiveness, low mental health literacy, low adherence, high drop-out, high severity of symptoms, difficulty in risk monitoring, low treatment engagement, difficulties in establishing a therapeutic relation, and limited facilities for computerized treatment are considered as barriers.

Overall, the findings of this review suggest that adolescents receiving unguided cCBT, guided cCBT, blended CBT, or self-help CBT-based computer game show a decrease in depressive symptoms after the intervention. Studies showed that symptom reduction was larger after unguided cCBT when compared to psychoeducation, larger in guided cCBT as compared to treatment as usual, and larger in blended CBT when compared to guided cCBT and face-to-face CBT [48,49]. These findings suggest that depressive symptoms reduce with all forms of cCBT including blended CBT. However, these findings need to be interpreted with caution, because they are based on a low number of studies; some without comparison to a control condition. Additionally, it needs to be taken into account that effects, especially of the unguided treatment, can be slightly overestimated due to the influence of research activities (e.g., administrative contact about questionnaires or therapist contact about severity of symptoms). It is known from earlier research in adults that effect sizes of computerized treatment increase when there is more support [66,67]. Especially in the treatment forms in which there is usually none to very little contact with therapist, this might have influenced the effects.

Intervention adherence to unguided cCBT (i.e., 62–94%), guided cCBT (i.e., 61–70%), blended CBT (i.e., 90%), and self-help CBT-based computer game (i.e., 60–81%) was comparable. The drop-out rates were also comparable between interventions, that is, 0–21% in unguided CBT, 17% in guided CBT, 0–13% in blended CBT. These percentages are comparable to the 14.2% drop-out of face-to-face psychotherapy for depressed adolescents reported by Watanabe, Hunot, Omori, Churchill, and Furukawa [14]. Again, we need to consider the impact of research activities, because it is known that drop-out in adult treatment is lower when there is more support during computerized treatment [66]. Therefore, the drop-out number might be an underestimation, especially in unguided computerized programs.

Mental health professionals reported that the therapeutic relation with adolescents was not negatively impacted when treatment is computerized. This is corresponds to earlier findings that online communication is an easy way for youth to connect to others and form new relationships [68,69]. However, mental health professionals qualified computerized interventions, whether or not guided or blended, as inadequate for adolescents with severe depression. The major concern is that mental health professionals are not able to monitor risk on self-harm or suicidal ideation. The intensive monitoring of risks is in accordance with the treatment guidelines for depression in youth [70]. Mental health professionals are supposed to make a safety plan when a patient suffers from suicidal ideation and this might, therefore, lead to a strong preference for face-to-face treatment in mental health professionals.

When considering acceptability, computerized and blended treatment were rated by participants as useful, helpful, easy to use, relatable, worth the time, and participants were satisfied. Privacy was mentioned as a benefit of unguided CBT. The participants attributed the improvements after blended CBT to the face-to-face sessions. 

The results show that depressed adolescents have low intention to seek help; they are more intended to not seek help than to get any form of treatment. A minority of adolescents would prefer computerized treatment. However, higher mental health literacy increased the intention to seek help. Adolescents stated that especially online interventions are accessible at any time and place, and give the possibility to be more easily tailored to their preferences. Further, studies show that mental health literacy increased in adolescents who received guided cCBT, blended CBT, or self-help CBT-based computer game. This indicates that computerized treatment might be an important part in a stepped care approach, where the adolescents first receive computerized treatment to increase mental health literacy, move on to blended treatment when symptoms remain, and only move to a more intensive face-to-face treatment if necessary, as was suggested before by Van Straten, et al. [71]. This approach offers accessible and efficient mental health support for depressed adolescents in an effective way.

Further, training for therapists in the computerized part of the protocol is an important necessity according to therapists. However, a study among psychologists regarding the use of internet-based therapeutic interventions showed that psychologists have little interest in using or receiving training in computerized therapy [29]. This discrepancy between knowing that training is important, and low interest in receiving training might explain the low use of computerized treatment in treating depressed youth.

Summarizing, evidence for effectiveness, mental health literacy, adherence, drop-out, the possibility to tailor the intervention, and forming a therapeutic relation can be seen as facilitators in using computerized treatment. The intention to seek help, symptom severity, acceptability, and facilities of computerized treatment can be seen as barriers. Risk monitoring is not possible in unguided treatment, but it can be done in guided or blended treatment. Time that is spent by therapists is nonexistent in unguided treatment. However, in guided or blended treatment, the time that is spent by therapist is comparable to face-to-face treatment. This shows that adolescents’ acceptability for computerized treatment and time that is spent by the therapist, both often mentioned as facilitators, appear to be barriers based on our research findings. Further, evidence of effectiveness, adherence, drop-out, and therapeutic relation were thought to be barriers for computerized treatment, and our findings showed that they are facilitating computerized and blended treatment.

### 4.1. Strengths and Limitations

This study has important limitations that need to be taken into account when interpreting the results. First, the studies included in this review were not selected on the study design, which resulted in studies with pre-post design with or without control condition, randomized controlled trials, cross-sectional studies, and studies with a qualitative study design. The quality of the designs is sometimes insufficient for examining effectiveness. However, not all included studies were aimed at effectiveness and, therefore, they give insight in other factors that could contribute to or withhold from using computerized or blended treatment in routine care. Second, findings are based on a small number of studies. Additionally, most participants were actively recruited to participate in the study, therefore characteristics, symptom level, and severity differed largely across the included studies. It is uncertain whether findings will hold when computerized or blended treatments are implemented in routine care [72,73]. Therefore, the findings need to be interpreted with caution and conclusions need to be carefully drawn. Third, we noticed that the terms guided computerized treatment and blended treatment were not used consistently across different studies. We choose to define treatment as blended only when treatment existed of computerized content through which patients work independently and patients receive guidance by their therapist by means of personal face-to-face contact, in line with the definition of Mathiasen, Andersen, Riper, Kleiboer, and Roessler [23] and Erbe, Eichert, Riper, and Ebert [24]. 

### 4.2. Suggestions for Future Research

There is some evidence regarding the effectiveness of computerized treatments for depressed youth, but evidence on effectiveness on blended treatment is scarce. Both treatment forms need to be examined in routine care to study whether the effects hold when they are implemented in routine care. Especially studies on the effectiveness of computerized and blended treatment in routine care can contribute to increase knowledge of mental health professionals and lower the threshold to use computerized, blended, or face-to-face treatment.

Furthermore, there is a gap of knowledge regarding the cost-effectiveness of computerized as well as blended treatment; hence, more research in this area is warranted. It has been suggested that blended care could reduce costs in comparison to face-to-face treatment. To our knowledge, only a limited number of studies assessed cost-effectiveness of blended treatment for depressed adults [74,75] and one review with 12 included studies was performed on the cost-effectiveness of internet and mobile-based interventions for treatment and the prevention of depression in adults [76]. However, no studies have been done on cost-effectiveness for depressed youth.

Lastly, the adherence rates and drop-out rates were found to be equal for computerized and face-to-face treatment [14]. Possibly, the adherence and drop-out rates might improve when individual patients are offered their preferred treatment. Moreover, studies on personalized treatment have shown that treatment effects can be improved when patients are assigned to treatment that is optimal for their characteristics (e.g., symptoms severity, life events, comorbidity) based on prediction models (e.g., [77,78]). 

## 5. Conclusions

Based on this review, we conclude that computerized and blended treatments seem promising as treatments for depressed youth in terms of effectiveness, adherence, drop-out, and forming a therapeutic relation. Additionally, the improvement in mental health literacy and the possibility to tailor the intervention can be seen as facilitators in using computerized treatment. However, adolescents’ intention to seek help, symptom severity, and facilities of computerized treatment can be seen as barriers. Further, adolescents’ acceptability for computerized treatment and time spent by therapist were thought to be facilitators, both often mentioned as facilitators, appear to be barriers that are based on our research findings. Highlighting the importance to monitor risks of severe depressive symptoms and possible suicidal ideation, unguided computerized treatment might be more appropriate for patients with mild to moderate depressive symptoms, where blended treatment could serve as a following step in the stepped care approach. Nonetheless, it needs to be noted that further research needs to be done to strengthen evidence for the use of computerized and blended treatment in routine care.

## Figures and Tables

**Table 1:** Characteristics of the included studies.

Study	Country	Intervention	Target Population	Study Participants	Study Type	Design	Outcome Measure
Abeles et al. (2009) [30]	United Kingdom	Unguided computer-based CBT	Youth (12–16 years) with depressive disorders	^a^*N* = 23	Effect evaluation	Pre-post design without control condition	Intervention effects
Berg et al. (2019) [39]	Sweden	Guided internet-based CBT vs. monitoring and non-specific counseling	Youth (15–19 years) with depressive disorders	^a^*N* = 70	Effect evaluation	RCT	Mental health literacy
Bobier et al. (2013) [50]	New Zealand	Self-help CBT-based computer game	Youth (16–19 years) admitted for severe psychiatric disorder (among which depressive disorders)	^a^*N* = 20	Feasibility, usability and/or acceptability evaluation;	Pre-post design without control condition	Acceptability; adherence and dropout
Bradford & Rickwood (2014) [60]	Australia	N/a ^b^	Youth (15–19 years) with mood disorders	^a^*N* = 231	Evaluation of preferred modes of help seeking	Cross-sectional study	Acceptability; intention to seek help
Bradley et al. (2012) [31]	Canada	Unguided internet-based CBT	Youth (15–18 years) with depressive symptoms	^a^*N* = 13	Feasibility, usability and/or acceptability evaluation	Pre-post design without control condition	Acceptability; intention to seek help; tailoring the intervention
Cheek et al. (2014) [51]	Australia	Self-help CBT-based computer game	Youth (12–19 years) with depressive symptoms ^c^	Youth (13–18) recruited from the general population *N* = 16	Feasibility, usability and/or acceptability evaluation	Pre-post design without control condition	Acceptability
Davidson et al. (2014) [32]	United States	Unguided internet-based behavioral activation	Youth at risk for post-disaster mental health problems among which mood disorders ^c^	Study 1: Youth (12–17 years) recruited from the general population *N* = 24 Study 2: ^a^ *N* = 291	Feasibility, usability and/or acceptability evaluation	Cross-sectional	Acceptability; treatment engagement; time spent; tailoring the intervention
De Vos et al. (2017) [46]	Netherlands	Blended CBT	Youth (12–18 years) with depressive disorders	^a^*N* = 32	Feasibility, usability and/or acceptability evaluation	Pre-post design without control condition	Acceptability; adherence and dropout; treatment engagement; time spent
Fleming et al. (2012) [52]	New Zealand	Self-help CBT-based computer game vs. wait list control	Youth (13–16 years) with depressive symptoms	^a^*N* = 32	Effect evaluation	Pragmatic RCT	Intervention effects; adherence and dropout; intention to seek help; risk monitoring
Forchuk et al. (2016) [58]	Canada	Internet-based monitoring tool	Youth (16–21 years) in mental health care with depressive disorders	Mental health care providers from acute and tertiary care facilities *N* = 9	Feasibility, usability and/or acceptability evaluation	Qualitative study	Acceptability; treatment engagement; time spent; therapeutic relation; symptoms severity
Johnston et al. (2014) [40]	Australia	Therapist-guided internet-based CBT	Young adults (18–24 years) with mild or moderate depressive symptoms	^a^*N* = 18	Effect evaluation	Pre-post design without control condition	Intervention effects; acceptability; adherence and dropout
Kobak et al. (2015) [47]	United States	Blended CBT vs. treatment as usual	Youth (12–17 years) with mood disorders	^a^*N* = 76	Feasibility, usability and/or acceptability evaluation	RCT	Intervention effects; acceptability; adherence and dropout; therapeutic relation; mental health literacy
Kurki et al. (2018) [41]	Finland	Guided internet-based intervention	Youth (15–17 years) with depressive or anxiety disorders	Registered nurses from outpatient clinics for adolescent psychiatry *N* = 9	Feasibility, usability and/or acceptability evaluation	Qualitative study	Symptoms severity; risk monitoring
Kurki et al. (2011) [61]	Finland	N/a ^b^	Youth (13–18 years) in mental health care with depressive symptoms	Registered nurses from outpatient clinics for adolescent psychiatry *N* = 14	Evaluation of preferred modes of help seeking	Qualitative study	Therapeutic relation; symptoms severity; facilities for computerized interventions
Logsdon et al. (2018) [33]	United States	Unguided internet-based intervention vs. no intervention control	Adolescent mothers (12–21 years) with depressive symptoms	^a^*N* = 292	Effect evaluation	Pre-post design with control condition	Intervention effects; acceptability; intention to seek help
Lokkerbol et al. (2018) [62]	Netherlands	N/a ^b^	Young adults and adults (from age 18) with depressive disorders	^a^*N* = 165	Evaluation of preferred modes of help seeking	Cross-sectional study	Acceptability
Lucassen Hatcher et al. (2015) [53]	New Zealand	Self-help CBT-based computer game	Youth (13–19 years) from sexual minorities with depressive symptoms	^a^*N* = 25	Feasibility, usability and/or acceptability evaluation	Qualitative study	Acceptability
Lucassen et al. (2013) [54]	New Zealand	Self-help CBT-based computer game	Youth (16–21 years) from sexual minorities with depressive symptoms	^a^*N* = 10	Feasibility, usability and/or acceptability evaluation	Qualitative study	Acceptability
Lucassen, Merry et al. (2015) [55]	New Zealand	Self-help CBT-based computer game	Youth (13–19 years) from sexual minorities with depressive symptoms	^a^*N* = 21	Feasibility, usability and/or acceptability evaluation	Pre-post design without control condition	Intervention effects; acceptability; adherence and dropout; mental health literacy
Lucassen et al. (2018) [56]	New Zealand	Self-help CBT-based computer game	Youth (15–21 years) from sexual minorities with depressive symptoms	LGBT+ young people *N* = 21 Professionals in health and social care *N* = 6	Feasibility, usability and/or acceptability evaluation	Qualitative study	Acceptability
Merry et al. (2012) [57]	New Zealand	Self-help CBT-based computer game	Youth (12–19 years) with depressive symptoms	^a^*N* = 187	Effect evaluation	RCT	Intervention effects; acceptability; adherence and dropout
O’Kearney et al. (2006) [42]	Australia	Teacher-guided internet-based CBT vs. standard personal developmental activities	Male youth (15–16 years) with depressive symptoms	^a^*N* = 87	Effect evaluation	Pre-post design with control condition	Intervention effects
Rickhi et al. (2015) [34]	Canada	Unguided spirituality informed internet-based intervention vs. wait list control	Youth (13–18 years) and young adults (19–24 years) with depressive disorders	^a^*N* = 31 and *N* = 31	Effect evaluation	RCT	Intervention effects; adherence and dropout
Sethi (2013) [48]	Australia	Unguided internet-based CBT vs. face-to-face CBT vs. blended CBT vs. no intervention control	Young adults (18–25 years) with mild or moderate depressive or anxiety symptoms	^a^*N* = 89	Feasibility, usability and/or acceptability evaluation	RCT	Intervention effects; adherence and dropout
Sethi et al. (2010) [49]	Australia	Unguided internet-based CBT vs. face-to-face CBT vs. blended CBT vs. no intervention control	Youth and young adults (15–25 years) with mild or moderate depressive or anxiety symptoms	^a^*N* = 38	Effect evaluation	RCT	Intervention effects
Smith et al. (2015) [35]	United Kingdom	Unguided computer-based CBT vs. wait list control	Youth (12–16 years) with depressive symptoms	^a^*N* = 112	Effect evaluation	RCT	Intervention effects
Stallard et al. (2011) [43]	United Kingdom	Psychology assistant-guided computer-based CBT vs. wait list control	Youth (11–16 years) with depressive or anxiety disorders	^a^*N* = 20	Feasibility, usability and/or acceptability evaluation	RCT	Intervention effects; acceptability; adherence and dropout; therapeutic relation
Stasiak et al. (2014) [36]	New Zealand	Unguided computer-based CBT game vs. computer-based psychoeducation	Youth (13–18 years) with depressive symptoms	^a^*N* = 34	Effect evaluation	RCT	Intervention effects; acceptability; adherence and dropout
Sundram et al. (2017) [59]	New Zealand	Internet-based monitoring tool	Youth (12–19 years) with mild or moderate depressive symptoms	Youth (12–19 years) *N* = 29 Clinicians (GPs and school’s health staff) *N* = 50	Feasibility, usability and/or acceptability evaluation	Qualitative study	Acceptability; treatment engagement; time spent; therapeutic relation; risk monitoring
Topooco et al. (2018) [44]	Sweden	Guided internet-based CBT vs. monitoring and non-specific counseling	Youth (15–19 years) with depressive disorders	^a^*N* = 70	Effect evaluation	RCT	Intervention effects; adherence and dropout; treatment engagement; time spent; therapeutic relation
Van der Zanden et al. (2012) [45]	Netherlands	Guided internet-based CBT vs. wait list control	Youth (16–25 years) with depressive symptoms	^a^*N* = 244	Effect evaluation	RCT	Intervention effects; adherence and dropout
Vangberg et al. (2012) [37]	Norway	Unguided internet-based CBT	Youth (15–20 years) with depressive symptoms	^a^*N* = 1239	Feasibility, usability and/or acceptability evaluation	Cross-sectional study	Acceptability
Wright et al. (2017) [38]	United Kingdom	Unguided computer-based CBT vs. self-help websites	Youth (12–18 years) with depressive symptoms	^a^*N* = 91	Effect evaluation	RCT	Intervention effects; adherence and dropout

^a^ Study participants were a sample from the target population; ^b^ Study was not aimed at a specific intervention, therefore, no specific target population is formulated; ^c^ Target population of the intervention was not used as study population; CBT: Cognitive Behavioural Therapy; RCT: Randomized Controlled Trial.
